# HA-1H T-Cell Receptor Gene Transfer to Redirect Virus-Specific T Cells for Treatment of Hematological Malignancies After Allogeneic Stem Cell Transplantation: A Phase 1 Clinical Study

**DOI:** 10.3389/fimmu.2020.01804

**Published:** 2020-08-20

**Authors:** Peter van Balen, Inge Jedema, Marleen M. van Loenen, Renate de Boer, H. M. van Egmond, Renate S. Hagedoorn, Conny Hoogstaten, Sabrina A. J. Veld, Lois Hageman, P. A. G. van Liempt, Jaap-Jan Zwaginga, Pauline Meij, H. Veelken, J. H. F. Falkenburg, Mirjam H. M. Heemskerk

**Affiliations:** ^1^Department of Hematology, Leiden University Medical Center, Leiden, Netherlands; ^2^Center for Clinical Transfusion Research, Sanquin Research, Leiden, Netherlands; ^3^Department of Immunohematology and Blood Transfusion, Leiden University Medical Center, Leiden, Netherlands; ^4^Department of Clinical Pharmacy and Toxicology, Leiden University Medical Center, Leiden, Netherlands

**Keywords:** HA-1, TCR gene transfer, minor histocompatibility antigen, allogeneic stem cell transplantation, graft-vs.-tumor effect

## Abstract

Graft-vs.-leukemia (GVL) reactivity after HLA-matched allogeneic stem cell transplantation (alloSCT) is mainly mediated by donor T cells recognizing minor histocompatibility antigens (MiHA). If MiHA are targeted that are exclusively expressed on hematopoietic cells of recipient origin, selective GVL reactivity without severe graft-vs.-host-disease (GVHD) may occur. In this phase I study we explored HA-1H TCR gene transfer into T cells harvested from the HA-1H negative stem-cell donor to treat HA-1H positive HLA-A^*^02:01 positive patients with high-risk leukemia after alloSCT. HA-1H is a hematopoiesis-restricted MiHA presented in HLA-A^*^02:01. Since we previously demonstrated that donor-derived virus-specific T-cell infusions did not result in GVHD, we used donor-derived EBV and/or CMV-specific T-cells to be redirected by HA-1H TCR. EBV and/or CMV-specific T-cells were purified, retrovirally transduced with HA-1H TCR, and expanded. Validation experiments illustrated dual recognition of viral antigens and HA-1H by HA-1H TCR-engineered virus-specific T-cells. Release criteria included products containing more than 60% antigen-specific T-cells. Patients with high risk leukemia following T-cell depleted alloSCT in complete or partial remission were eligible. HA-1H TCR T-cells were infused 8 and 14 weeks after alloSCT without additional pre-conditioning chemotherapy. For 4/9 included patients no appropriate products could be made. Their donors were all CMV-negative, thereby restricting the production process to EBV-specific T-cells. For 5 patients a total of 10 products could be made meeting the release criteria containing 3–280 × 10^6^ virus and/or HA-1H TCR T-cells. No infusion-related toxicity, delayed toxicity or GVHD occurred. One patient with relapsed AML at time of infusions died due to rapidly progressing disease. Four patients were in remission at time of infusion. Two patients died of infections during follow-up, not likely related to the infusion. Two patients are alive and well without GVHD. In 2 patients persistence of HA-1H TCR T-cells could be illustrated correlating with viral reactivation, but no overt *in-vivo* expansion of infused T-cells was observed. In conclusion, HA-1H TCR-redirected virus-specific T-cells could be made and safely infused in 5 patients with high-risk AML, but overall feasibility and efficacy was too low to warrant further clinical development using this strategy. New strategies will be explored using patient-derived donor T-cells isolated after transplantation transduced with HA-1H-specific TCR to be infused following immune conditioning.

## Introduction

Allogeneic hematopoietic stem cell transplantation (alloSCT) is used to induce or consolidate long-term remissions in patients with hematological malignancies. Although chemotherapy and/or irradiation is part of the essential conditioning treatment to allow engraftment of donor hematopoietic cells, the advantage of alloSCT over high dose chemotherapy or autologous stem cell transplantation is the potential profound effect of the alloimmune response mediated by donor T cells resulting in eradication or persistent control of the malignant hematopoietic clone in the patient (1). After HLA-matched alloSCT, this graft vs. leukemia (GVL) reactivity is mainly mediated by donor T cells recognizing minor histocompatibility antigens (MiHA) on recipient hematopoietic cells (2–6). If donor T cells recognize MiHA expressed on both recipient hematopoietic and non-hematopoietic cells, GVL reactivity is frequently accompanied by graft-vs.-host disease (GVHD) (7–10). If MiHA are targeted that are exclusively expressed on hematopoietic cells of recipient origin, selective GVL reactivity will occur coinciding with conversion to full donor chimerism of the hematopoietic system (11). Several MiHA have been reported to be selectively expressed on hematopoietic cells, and may therefore be targets for a specific GVL reactivity (12, 13). Although various factors may influence the balance between GVL and GVHD reactivity including dosing and timing of donor T-cell infusion, predictable selective induction of GVL reactivity appears to require infusion or induction of donor T cells that specifically target antigens that are selectively expressed on (malignant) hematopoietic cells of the patient (1, 14–17).

HA-1H is a MiHA with a population frequency of 30% which is selectively presented by the restriction allele HLA-A^*^02:01 on cells from hematopoietic origin, which can be recognized by T cells from HLA-A^*^02:01 positive individuals homozygous for the allelic counterpart HA-1R, lacking HA-1H (18, 19). We have illustrated that HA-1H-specific T cells can be found in the majority of homozygous HA-1R HLA-A^*^02:01 positive donors (20). We have demonstrated that donor T cells recognizing HA-1H can contribute to a specific GVL reactivity in the absence of severe GVHD (21). However, direct purification of HA-1H-specific T cells from donor peripheral blood to be used as therapeutic reagent has been difficult to achieve due to low frequencies. We therefore previously attempted to expand HA-1H specific T-cell lines using an *in-vitro* culture protocol. Although we have demonstrated that HA-1H-specific T-cell lines could be generated and infused into patients without toxicity, *in-vivo* expansion and clinical benefit could not be illustrated (20).

T-cell receptor (TCR) gene transfer appears to be an attractive *in-vitro* strategy to generate large numbers of antigen specific T cells that can be used for adoptive transfer. Autologous T cells modified to induce a TCR targeting an antigen of choice have been demonstrated to have clinical effectiveness after transfer into patients with solid tumors (22–25). Based on these encouraging results, we hypothesized that donor T cells engineered to express an HA-1H-specific TCR may be used to eliminate patient hematopoiesis including the malignant clone in HA-1H positive patients transplanted with an HA-1H negative (homozygous HA-1R positive) donor. Since unselected donor T cells may induce GVHD when infused into patients after alloSCT, we hypothesized that engineering virus-specific T cells from donor origin to express the HA-1H TCR would create a therapeutic product unlikely to induce GVHD. We and others have illustrated that the infusion of virus-specific T cells from donor origin into patients after alloSCT can have a profound anti-viral reactivity without toxicity (26–32). In addition, virus-specific T cells engineered to coexpress tumor-specific receptors demonstrated improved persistence after treatment of individuals with neuroblastoma (33). Therefore, T cells harboring both the endogenous virus-specific TCR and the transferred HA-1H TCR may have both beneficial specificities. To ensure appropriate expression of the HA-1H TCR in the virus-specific T cells and limit the risk of miss-paired dimerization between the endogenous and exogenous TCR, we used a codon optimized cysteine modified TCR, in which the TCR-α and -β chains were linked by a T2A sequence (34). The good manufacturing practice (GMP) grade production of HA-1H TCR transduced virus-specific cells for this HA-1H TCR gene therapy study was established by using MHC-I-Streptamer-based isolation technology and subsequent transduction with the HA-1H TCR using retroviral vectors (35).

In this phase I clinical study we explored the feasibility to generate HA-1H TCR gene transduced CMV or EBV-specific T cells harvested from the stem cell donor to obtain larger numbers of HA-1H-specific T cells and treat HLA-A^*^02:01 positive HA-1H positive patients with hematological malignancies, and evaluated potential toxicity and efficacy. After prophylactic infusion of HA-1H TCR-transduced CMV or EBV-specific T cells 8 and 14 weeks after T-cell depleted alloSCT with prescheduled postponed donor lymphocyte infusion (DLI) 6 months after alloSCT (17, 36), no infusion-associated toxicity, delayed toxicity, or GVHD was observed. In addition, persistence or expansion of HA-1H TCR transduced T cells was observed in 3 out of 5 patients. However, overall feasibility and efficacy was too low to allow further development of this specific therapeutic product. New strategies will be explored to evaluate potential efficacy of HA-1H TCR T-cells to control recurrence of hematological malignancies of HLA-A^*^02:01 positive HA-1H positive patients transplanted with an HA-1H negative donor.

## Materials and Methods

### Generation of HA-1H TCR-Transduced CMV or EBV-Specific T Cells

HA-1H TCR-transduced CMV and/or EBV-specific T-cell products were generated as described previously (35). In short, either one or two virus-specific T-cell populations were isolated from a donor leukapheresis product harvested prior to the G-CSF mobilization and cryopreserved until further use. Virus-specific T-cell populations were isolated using the MHC-I-*Strep*tamer isolation technology (Juno Therapeutics GmbH, a Celgene company (formerly Stage Therapeutics), Munich, Germany). Isolation complexes (MHC-I-*Strep*tamers) were generated per T-cell specificity by incubation of peptide-loaded MHC-I-*Strep*-tag fusion proteins with magnetically labeled *Strep*-Tactin (*Strep*-Tactin nanobeads). The pool of MHC-I-*Strep*tamers was incubated with 1–2 × 10^9^ donor PBMC for 45 min at 4°C. MHC-I-*Strep*tamer-bound cells were isolated using a CliniMACS Plus instrument (Miltenyi Biotec, Bergisch Gladbach, Germany) according to the manufacturer's instructions. MHC-I-*Strep*tamers were dissociated from the positively isolated cells using 1 mM of D-Biotin. Selected virus-specific T cells were cultured with irradiated (25 Gy) autologous peripheral blood mononuclear cells (PBMNC) as feeder cells (ratio 1:5) in T-cell culture medium consisting of Iscove's Modified Dulbecco's Medium (IMDM; Lonza, Basel, Switzerland) supplemented with 10% human serum, 100 IU/mL IL-2 (Proleukin®/Aldesleukin, Novartis, Arnhem, Netherlands) and 10 ng/mL interleukin-15 (Cellgenix, Freiburg, Germany). The virus-specific T cells were transduced 2–3 days after isolation with GMP-grade retroviral supernatant encoding the HA-1H TCR (EUFETS GmbH, Idar Oberstein, Germany) spun down on retronectin-coated (15 μg/well; Takara Bio, St-Germain-en-Laye, France) 24-wells clear flat-bottomed microplates (Greiner Bio-one, Alphen aan den Rijn, Netherlands) for at least 4 h at 37°C. The cells were subsequently cultured for 10–14 days in T-cell culture medium.

### Study Design

The study *Administration of HA-1H TCR-transduced virus-specific T cells after allogeneic stem cell transplantation in patients with high risk leukemia* was registered at www.clinicaltrialsregister.eu as EudraCT number 2010-024625-20. The study was approved by the central committee on research involving human subjects (CCMO), and the LUMC Institutional Review Board. From all patients and donors written informed consent was obtained. The primary objective of the study was to investigate the feasibility and safety of administration of donor derived HA-1H TCR-transduced virus-specific T cells after T-cell depleted alloSCT. Feasibility was defined as more than 50% of patients receiving at least one infusion of HA-1H TCR-transduced virus specific T cells posttransplant after inclusion in this study. Secondary objectives were to evaluate the persistence of HA-1H TCR-transduced virus-specific T cells after infusion, and to evaluate whether administration of HA-1H TCR-transduced virus-specific T cells makes patients eligible for standard donor lymphocyte infusion (DLI) at 6 months after alloSCT. Patients 18–75 years of age with high risk leukemia in complete or stable partial remission prior to transplant were eligible if they were HLA-A^*^02:01 and HA-1H positive, and transplanted with an HLA-matched HA-1H negative donor. The donor needed to be CMV and/or EBV seropositive allowing isolation of sufficient EBV or CMV-specific T cells. AlloSCT was performed as published previously (6, 17). HA-1H TCR-transduced virus-specific T cells were scheduled 8 and 14 weeks after alloSCT, since at that time points the alemtuzumab used in the conditioning regimen will not be circulating in the patient anymore. Contraindications for actual infusion of HA-1H TCR-transduced virus-specific T cells were acute GVHD overall grade II or higher or treatment with corticosteroids at a dose of 0.5 mg/kg prednisone or higher.

### Flow Cytometry

Absolute numbers of circulating CD4 T, CD8 T, B, and NK cells were determined by the clinical Laboratory for Specialized Hematology (LUMC) as part of routine immune monitoring after transplantation on anticoagulated fresh venous blood using Trucount tubes (BD, Becton Dickinson, Breda, Netherlands) following the manufacturer's instructions. Samples were stained with allophycocyanin (APC)-conjugated CD3, PacificBlue-conjugated CD4, fluorescein isothiocyanate (FITC)-conjugated CD8, APC-H7-conjugated CD14, R-phycoerythrin (PE)-conjugated CD16, PE-Cy7-conjugated CD19, V500-conjugated CD45, and PE-conjugated CD56 (all from BD) antibodies and analyzed using a FACSCanto (BD).

Thawed PBMCs and bone marrow MNC cells from immunomonitoring samples and T-cell lines and clones were analyzed for binding to HA1H and virus-specific pMHC-tetramers by staining with PE- or APC-labeled pMHC-tetramers, and an Alexa700-conjugated antibody against CD8 (Invitrogen/Calteg, Buckingham, United Kingdom) combined with FITC-labeled antibodies against CD4, CD14, and CD19 (BD Pharmingen, San Jose, California). PBMCs and T-cell lines or clones were first incubated with 2 μg/ml pMHC-tetramers for 15 min at 37 °C before antibodies were added and incubated for an additional 15 min at 4°C. These analyses were performed on an LSRII (BD Biosciences, Franklin Lakes, New Jersey) and analyzed using Diva Software (BD Biosciences) or FlowJo Software (TreeStar, Ashland, Oregon).

### Vector-Specific Real-Time Quantitative PCR of HA-1H TCR

Genomic DNA was isolated either using the AllPrep DNA/RNA/Protein mini kit (Qiagen) or QIAamp DNA Micro Kit (Qiagen) following manufacturer's instructions. Samples were run on a 7900HT RT-PCR System of Applied Biosystems. The following vector-specific HA1 TCR primers were used; forward primer resides in the optimized constant domain of the beta chain 5′ CTGTACGCCGTGCTGGTG 3′, reverse primer resides in the T2A region 5′ GGGATTCTCCTCCACGTCACC 3′ and the antisense probe also resides in the T2A region 5′ TGTTAGAAGACTTCCTCTGCCCTC 3′. The Probe used VIC as dye and TAMRA as quencher. Each sample was run in duplicate with 200 ng genomic DNA per well (qPCR core kit Eurogentec) at 65°C for 50 cycles.

### T-Cell Reactivity Assays

T cells were cultured in T-cell medium (TCM) consisting of IMDM supplemented with 5% heat-inactivated FBS (Gibco, Thermo Fisher Scientific), 5% human serum, 3 mM l-glutamine (Lonza), 100 U/ml penicillin/streptomycin (Lonza), and 100 IU/ml IL-2 (Novartis). T-cell recognition was measured by IFN-γ ELISA (Sanquin) according to manufacturer's instructions. T cells (5,000 cells) were co-cultured with 30,000 cells (LCLs) or 60,000 cells (primary cells) in 60 μl TCM per 384 well flat bottom plates (Greiner Bio-One). Supernatants were harvested after overnight incubation to measure IFN-γ release. In peptide-pulsed conditions, stimulator cells were preincubated with 1 μM of the relevant peptide for 30 min at room temperature before addition to T cells.

### Generation of T-Cell Clones

Virus-specific T cells were isolated from frozen PBMCs via FACS sorting using PE-labeled pMHC-tetramers. T cells were initially incubated with PE-labeled pMHC-tetramers for 1 h at 4°C and subsequently incubated with anti-CD8-FITC for 20 min at 4°C. pMHC-tetramer^+^ CD8^+^ T-cells were single-cell sorted into round-bottom 96-well plates containing 5 × 10^4^ irradiated (35 Gy) allogeneic PBMNC as feeders in 100 μl T-cell medium supplemented with 0.8 μg/ml phytohemagglutinin (PHA; Oxoid Limited, Basingstoke, UK).

## Results

### Feasibility and Characteristics of HA-1H TCR-Transduced CMV or EBV-Specific T-Cell Products

Nine donors were approached and gave consent to undergo leukapheresis to obtain PBMC for the generation of HA-1H TCR-transduced CMV or EBV-specific T-cell products. 2 donors were seropositive for both EBV and CMV, whereas 7 donors were only EBV seropositive. In 3 of these 7 donors frequencies of EBV-specific T cells with the appropriate specificity as measured by pMHC-tetramers were not above background, and therefore no EBV-specific T cells could be harvested, and no cell lines could be generated. From 6 donors, attempts were made to generate HA-1H TCR-transduced CMV or EBV-specific T cells (Table 1). From 5 donors a total of 10 HA-1H TCR-transduced CMV or EBV-specific T-cell products fulfilling the release criteria were generated (Table 1). Total cell numbers ranged from 3 to 283 × 10^6^ comprised of 96–99% T cells containing 74–100% virus-specific T cells and 11–41% HA-1H TCR-transduced T cells as measured by specific pMHC-tetramer staining. From donor UPG (patient 9) only very low numbers of EBV-specific T cells could be isolated, and at the end of the culture only 0.16 × 10^6^ T cells were retrieved with only 5% of HA-1H TCR T cells, and therefore this product did not fulfill the release criteria. More detailed data about the composition of generated T-cell products is depicted in Supplemental Figure 1. These results illustrate that if virus-specific T cells could be detected in peripheral blood of the donors, in 5/6 cases HA-1H TCR- transduced CMV or EBV-specific T-cell products could be reproducibly generated.

**Table 1:** Characteristics of starting material and release specifications of generated products.

**Donor**	**EBV**	**CMV**	**Line**	**Streptamer**		**Starting material**			**Directly after isolation**			**End of culture**					
	**status**	**status**															
	**donor**	**donor**															
				**1**	**2**	**MNC**	**Strept**	**Strept**	**Cells**	**Strept**	**Strept**	**Cells**	**CD3**	**Strept**	**Strept**	**HA1-**	**Total**
							**1**	**2**		**1**	**2**			**1**	**2**	**TCR**	**tetramer**
						**(10^**6**^)**	**(%)**	**(%)**	**(10^**6**^)**	**(%)**	**(%)**	**(10^**6**^)**	**(%)**	**(%)**	**(%)**	**(%)**	**pos (%)**
001	pos	pos	1	CMV-pp50-A1-VTE	EBV-BZLF1-B8-RAK	1590	1.2	1.2	10.5	49	45	283	99	27	33	39	94
			2	CMV-pp50-A1-VTE	EBV-BZLF1-B8-RAK	1890	1.2	1.8	8.3	52	45	228	98	27	26	36	97
002	pos	neg	1	EBV-BMLF1-A2-GLC	–	2351	0.2	–	1.7	72	–	3.1	99	59	–	41	100
			2	EBV-BMLF1-A2-GLC	–	2341	0.2	–	0.6	n.d.	–	2.9	97	44	–	30	83
003	pos	neg	1	EBV-BMLF1-A2-GLC	EBV-BZLF1-B8-RAK	2272	0.05	0.53	7.6	2.6	49	101	97	13	30	30	74
			2	EBV-BMLF1-A2-GLC	EBV-BZLF1-B8-RAK	2024	0.04	0.55	3.8	2.8	52	15	97	15	32	18	78
004	pos	neg	1^*^														
005	pos	pos	1	CMV-pp65-A2-NLV	EBV-EBNA3A-B7-RPP	1480	0.04	0.07	0.8	17	36	88	99	4.5	56	35	97
			2	CMV-pp65-A2-NLV	EBV-EBNA3A-B7-RPP	985	0.03	0.05	0.22	n.d.	n.d.	54	96	3.6	58	33	98
006	pos	neg	1^*^														
007	pos	neg	1	EBV-EBNA3A-B7-RPP	EBV-BZLF1-B8-RAK	1826	0.1	1.5	10.4	8	84	44	99	58	9.3	11	97
			2^*^	EBV-EBNA3A-B7-RPP	EBV-BZLF1-B8-RAK	2000	0.1	1.4	4.6	10	87	21.6	99	64	15	13	99
008	pos	neg	1^*^														
009	pos	neg	1^*^	EBV-BMLF1-A2-GLC	–	2074	0.1	–	0.7	80	–	0.16	84	72	–	5	77

* *No infusion of HA1H TCR-transduced T cells*.

### Safety and Clinical Effect of Infusion of HA-1H TCR-Transduced CMV or EBV-Specific T Cells

Nine patients were included in the study. As illustrated in Table 1 for 4 patients (patients 4, 6, 8, and 9) no HA-1H TCR-transduced CMV or EBV-specific T-cell product could be generated. Characteristics of patients who received a product are depicted in Table 2.

**Table 2:** Characteristics of patients who received HA-1H TCR-transduced T cells.

**Study ID**	**001**	**002**	**003**	**005**	**007**
Age	51	36	65	47	51
Gender	Female	Female	Female	Female	Male
Disease	Therapy related AML	AML	AML	AML	B-LBL
Cytogenetics/molecular diagnostics	t (9,11) and t (1,15)	NCA	NPM1+ FLT3+	Monosomal karyotype	MLL+ t (4,11)
Number of infusions	2	2	2	2	1
Transplant manipulation	CD34 selection	Alemtuzumab	Alemtuzumab	Alemtuzumab	Alemtuzumab
Stem cell donor	MUD	Sibling	MUD	Sibling	MUD
Conditioning regimen	MA	NMA	NMA	MA	MA
Patient chimerism at first infusion (MNC-leucocytes-granulocytes)	0-0-0	1-1-?	0-0-0	0-0-0	0-0-0
Patient chimerism at second infusion (MNC-leucocytes-granulocytes)	0-0-0	1-1-2	0-0-0	83-77-21	
CMV load in serum at first infusion	3.2	0	0	2.5	0
CMV load in serum at second infusion	2.3	0	0	0	
Highest detectable CMV load in serum (weeks after first infusion)	4.5 (11)	0	0	2.4 (3)	0
EBV load in serum at first infusion	0	0	0	0	0
EBV load in serum at second infusion	0	0	0	0	
Highest detectable EBV load in serum (weeks after first infusion)	0	0	0	0	5.7 (7)
Development of GVHD (weeks after first infusion)	No	No	No	No	No
Infusion of standard care DLI 6 months after alloSCT	No	Yes	Yes	No	No
Adverse events (between first infusion and 6 months post alloSCT)	• Pulmonal aspergillus • Candidaemia • Parvovirus • Bacteriemia • S. Haemolyticus	None	None	Relapse AML	• PTLD • Pulmonal aspergillus
Duration of follow up in weeks after first infusion	19	234	224	19	7
Alive at last follow up	No	Yes	Yes	No	No
Cause of death	Multiple infections			Relapse AML	PTLD

*AML, acute myeloid leukemia; B-LBL, B-cell lymphoblastic leukemia; MUD, matched unrelated donor; MA, myelo-ablative; NMA, non-myelo-ablative; PTLD, post-transplant lymphoproliferative disease. NCA, no cytogenetic abnormalities*.

**Figure 1:**
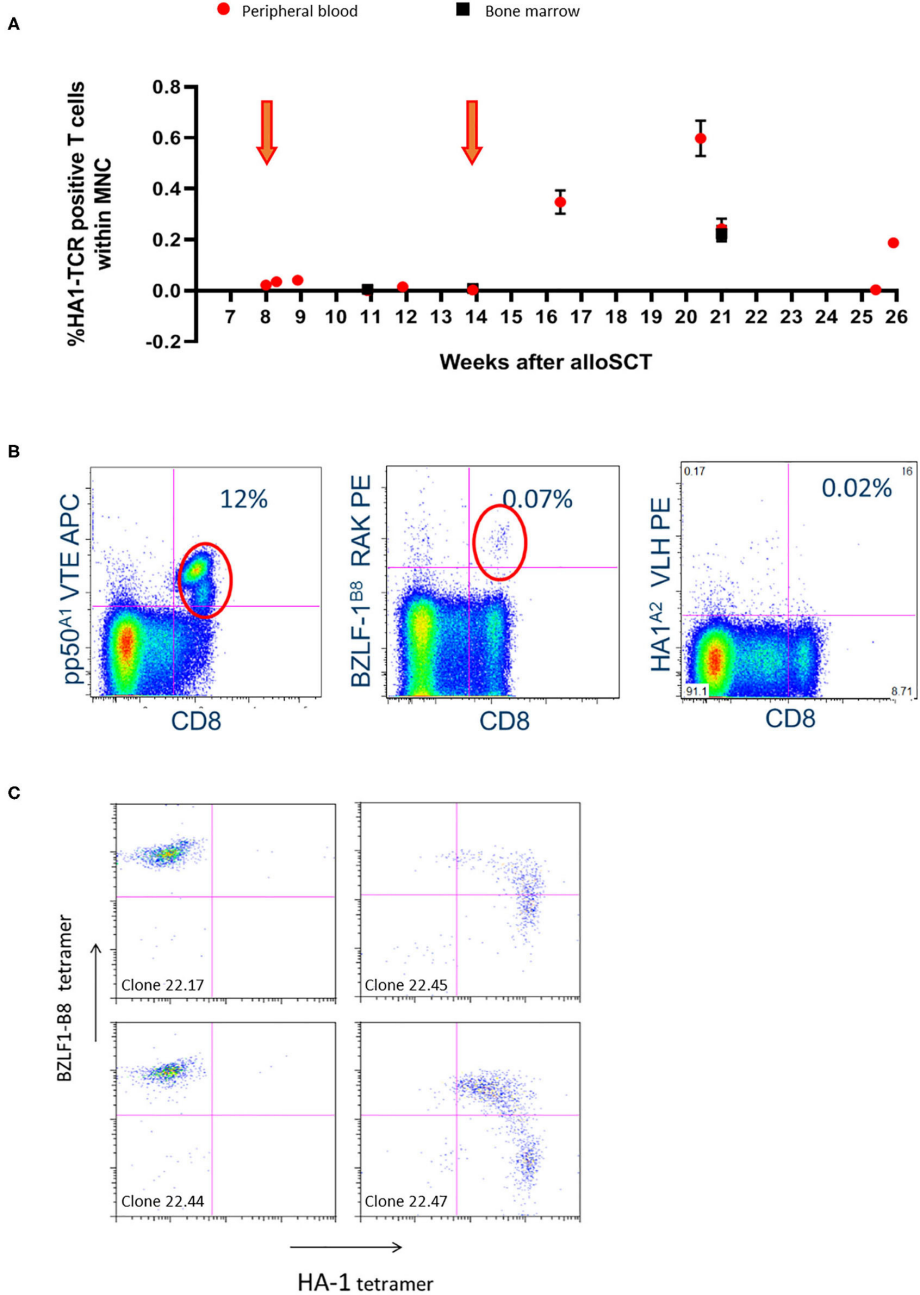
Significant *in-vivo* persistence of HA-1H TCR-transduced T cells could be observed during follow-up with evidence of expansion after the second infusion in patient 001. **(A)** Vector-specific qPCR analysis was performed on peripheral blood and bone marrow samples at indicated time-points. Six weeks after the second infusion the highest peak of HA-1H TCR-transduced CMV or EBV-specific T cells peripheral blood and bone marrow samples was detected. Orange arrows illustrate infusion of HA-1h TCR modified T cells. **(B)** Facs analysis was performed on peripheral blood sample 6 weeks after infusion of the second cell line. Low numbers of EBV-specific T cells were observed (0.07%), and high frequencies of CMV-specific T cells were found including the infused CMV-pp50-A1-VTE specificity (12%). **(C)** T cells were isolated from PBMCs 6 weeks after second infusion using EBV-BZLF1-B8-RAK tetramers, single cell sorted and expanded, and tested after 14 days with the different pMHC-tetramers indicated. 50% of the EBV-BZLF1-B8-RAK-specific T-cell clones expressed the HA-1H TCR as measured by pMHC-tetramer analysis.

Four patients received the 2 scheduled infusions of the HA-1H TCR-transduced CMV or EBV-specific T cells, whereas patient 7 only received one dose. No immediate transfusion-related side effects were observed. Patient 1 showed persistent lymphopenia from the time of transplant until the end of follow-up. Significant *in-vivo* persistence of HA-1H TCR-transduced T cells could be observed by vector-specific PCR analysis during follow-up with evidence of expansion after the second infusion (Figure 1A). The patient developed antibody-mediated neutropenia and thrombocytopenia 7 weeks after the infusion of the second cell line. Despite treatment with immunoglobulins, unmanipulated DLI, antibiotics and antifungal medication, the patient died from multiple viral, bacterial and fungal infections 19 weeks after the first infusion. In patients 2 and 3 no side effects occurred, no GVHD developed, and both patients received scheduled DLI 6 months after transplantation. At the time of last follow-up both patients are alive and well.

**Figure 2:**
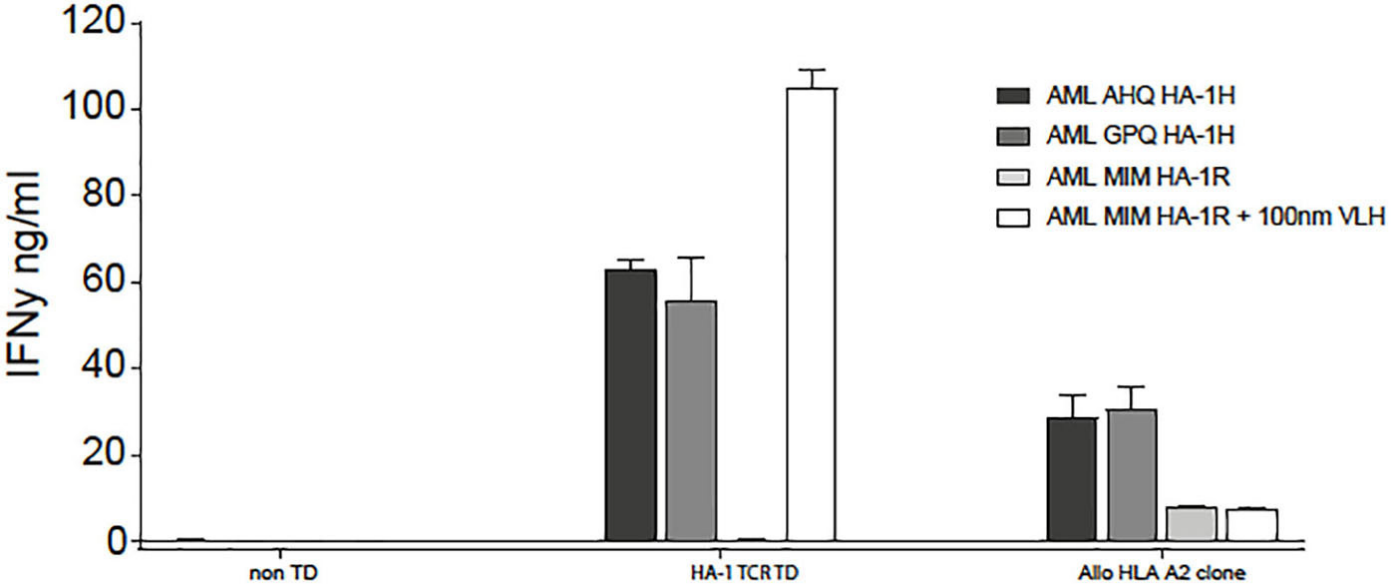
HA-1H TCR-transduced T cells recognized HLA-A*02:01 positive, HA-1H positive primary AML cells, both patient AML cells (AHQ) at the time of relapse, as well as third party HLA-A*02:01 positive, HA-1H positive AML (GPQ) cells. HLA-A*02:01 positive, HA-1H negative AML (MIM) cells were only recognized if HA-1H peptide (VLH) was exogenously loaded on the AML cells.

In patient 5, despite full donor chimerism as measured in peripheral blood, smoldering relapse was documented in the bone marrow aspirate at the time of the first infusion. The second infusion was performed as scheduled, but the patient developed progressive disease. Despite re-remission induction chemotherapy, no remission could be obtained, and the patient died from relapsed AML. No side effects related to the infusion were documented. Up to 3 weeks after the first and two weeks after the second infusion, low frequencies of HA-1H TCR-transduced CMV or EBV-specific T cells could be detected as measured by vector-specific PCR analysis, but no significant expansion of the cells could be documented (Table 3). This was not due to the inability of the relapsed AML to be targeted by HA-1H TCR-transduced CMV or EBV-specific T cells, since *in-vitro* analysis illustrated appropriate recognition of the leukemic cells (Figure 2) by these T cells.

**Table 3:** Results of PCR measurements of HA-1H TCR transduced virus-specific T cells to evaluate persistence.

**Weeks after 1st infusion**	**Weeks after 2nd infusion**	**HA-1H TCR positive T cells within total peripheral blood mononuclear cells (%)**				
		**Patient 001**	**Patient 002**	**Patient 003**	**Patient 005**	**Patient 007**
0	−6	Undetectable	Undetectable	Undetectable	Undetectable	Undetectable
1	−5	0.041	Undetectable	Undetectable	1 – 10^−7^	Undetectable
3	−3	0.001	Undetectable	Undetectable	3 – 10^−7^	1.70
6	0	0.003	Undetectable	Undetectable	Undetectable	0.40
8	2	0.348	Undetectable	Undetectable	3 – 10^−7^	0.52
12	6	0.598	Undetectable	Undetectable	Undetectable	
16	10	0.188	Undetectable	Undetectable	Undetectable	

**Figure 3:**
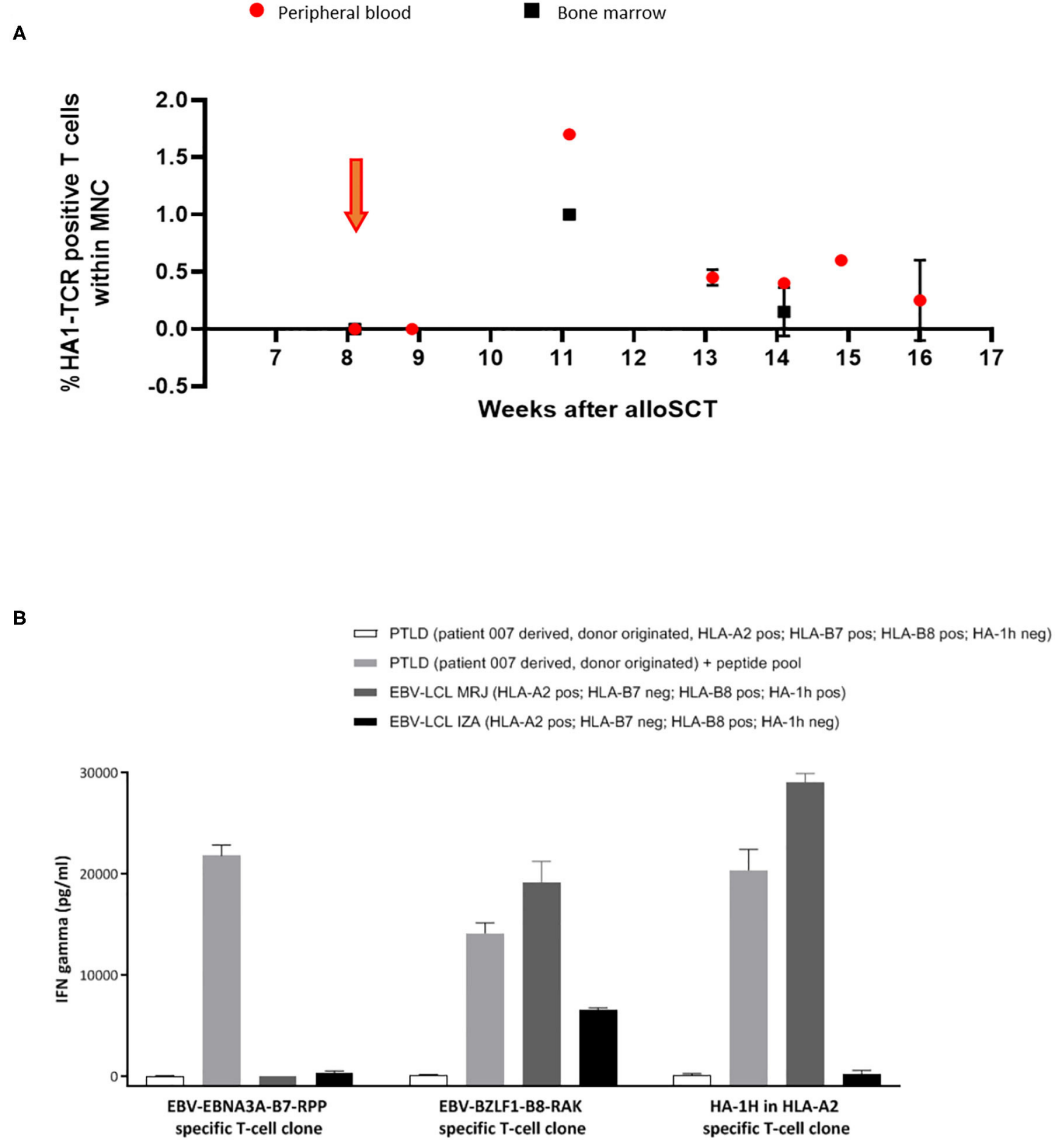
Significant expansion and persistence of HA-1H TCR-transduced T cells could be documented in peripheral blood and bone marrow samples of patient 007. **(A)** Vector -specific qPCR analysis was performed on peripheral blood and bone marrow samples at indicated time-points. Orange arrow illustrates infusion of HA-1H TCR-modified T cells. **(B)** EBNA-3A-B7 and BZLF1-B8 and HA1H specific T cells were stimulated with patient 007 PTLD (SLC PTLD) that was not loaded or loaded with pool of the 2 EBV peptides, LCL-MRJ (HLA-A2+, B7-, B8+, HA-1H+, BZLF1+), and LCL-IZA (HLA-A2+, B7-, B8+, HA-1H-, BZLF1+). PTLD of patient 007 was not recognized by EBV-specific T-cell populations unless they were exogenously loaded with EBV-specific peptides. This PTLD was also not recognized by HA-1H-specific T cells, because the PTLD was donor-derived and therefore HA-1H negative. PTLD sample consisted of monoclonal B cells after CD19 enrichment of PBMNC.

In patient 7, from 3 weeks after infusion, expansion and persistence of HA-1H TCR-transduced T cells could be documented by vector-specific PCR in peripheral blood and bone marrow samples (Figure 3A), which coincided with an EBV reactivation. Five weeks after the infusion, limited signs of skin GVHD were observed, completely resolving after application of topical steroids. One week later the patient was admitted to the hospital with high fever caused by EBV-associated posttransplant lymphoproliferative disease (PTLD) with massive B-cell expansion. Despite the presence of EBV-specific T cells in peripheral blood these virus-specific T cells did not further expand, and despite treatment with rituximab and steroids, the condition rapidly deteriorated and the patient died from severe systemic disease.

In summary, for 5 out of 9 included patients HA-1H TCR-transduced CMV or EBV-specific T-cell products could be successfully generated and infused. In 3 patients persistence or expansion of HA-1H TCR-transduced CMV or EBV-specific T cells was detected. No transfusion-related toxicity was observed, and no significant GVHD developed after infusion. Three of the 5 patients died from complications probably not related to the infusion.

### Persistence and Functionality of Infused HA-1H TCR-Transduced CMV or EBV-Specific T Cells

Significant *in-vivo* persistence and/or expansion of HA-1H TCR-transduced CMV or EBV-specific T cells was documented in patients 1 and 7 (Figures 1A, 3A). From patient 1, 6 weeks after the second infusion at the highest peak of HA-1H TCR-transduced CMV or EBV-specific T cells (Figure 1A) peripheral blood and bone marrow samples were isolated. Low numbers of EBV-specific T cells were observed, but probably due to CMV reactivation high frequencies of CMV-specific T cells were found including the infused CMV-pp50-A1-VTE specificity (Figure 1B). However, no significant frequencies of HA-1H TCR-transduced T cells were detected as measured by pMHC-tetramer analysis (Figure 1B). To analyze whether HA-1H TCR-transduced T cells were present within these virus-specific T-cell populations, the CMV-pp50-A1-VTE and EBV-BZLF-1-B8-RAK positive T cells were FACS sorted using pMHC-tetramers. Vector-specific PCR analysis on the CMV-pp50-A1-VTE tetramer positive sorted cells demonstrated that only a very low frequency of HA-1H TCR-transduced T cells (0.01%) could be identified within the CMV-specific population. Apparently, after triggering by CMV antigen, non-HA-1H TCR-transduced CMV-specific T cells preferentially had expanded. In contrast, 50% of the EBV-BZLF1-B8-RAK-specific T-cell clones expressed the HA-1H TCR as measured by pMHC-tetramer analysis (Figure 1C) and vector-specific PCR analysis (data not shown).

In patient 7, significant persistence and expansion of HA-1H TCR-transduced EBV-specific T cells was observed (Figure 3A), probably provoked by exposure to EBV antigen as documented by the presence of circulating EBV antigen by PCR analysis. Despite the presence of EBV-specific T cells rapidly progressive donor-derived monoclonal EBV-associated PTLD developed. To determine why this PTLD could escape control by the circulating EBV-specific T cells, we analyzed *in-vitro* whether the PTLD could be recognized by EBV-EBNA3A-B7-RPP or EBV-BZLF1-B8-RAK T-cell populations. As illustrated in Figure 3B, the PTLD was not recognized by these EBV-specific T-cell populations unless they were exogenously loaded with EBV-specific peptides. These results illustrate that the PTLD could escape control by the EBV-specific T cells due to absence of relevant endogenously processed viral antigens.

## Discussion

In this study we investigated whether HA-1H TCR-transduced CMV or EBV-specific T-cell products could be reproducibly generated from PBMCs of the stem cell donors seropositive for CMV or EBV. Our data illustrate that if sufficient virus-specific T cells could be isolated from the donor, HA-1H TCR-transduced CMV or EBV-specific T-cell products meeting the predefined release criteria could be generated. The drawback of the present study was that with the limited numbers of EBV or CMV- specific *Streptamer* products available, in several cases insufficient numbers of donor-derived virus-specific T cells could be harvested, especially in donors that were CMV seronegative implying that only EBV-specific T cells were available. This resulted in the successful production of the HA-1H TCR-transduced CMV or EBV-specific T-cell products ranging from 3 to 280 x 10^6^ antigen-specific T cells for only 5 of 9 patients included in the study. The 5 HLA-A^*^02:01 and HA-1H positive patients with AML who were treated with the HA-1H TCR-transduced CMV or EBV-specific T-cells 8 and 14 weeks after T-cell depleted alloSCT did not experience infusion related toxicity, and no significant development or worsening of GVHD was observed. The complications observed in the patients after the infusions were considered not likely to be caused by the investigational product since at the time of these complications no significant expansion of the infused HA-1H TCR-transduced CMV or EBV-specific T cells was observed. From these data we concluded that although the infusions appear to be safe, the overall feasibility and efficacy of the procedure was too low to warrant further developments of this specific investigational product.

Several reasons may underlie the lack of expansion directly after infusion of HA-1H TCR-transduced CMV or EBV-specific T cells. As demonstrated in clinical trials using CAR T cells for the treatment of relapsed or refractory hematological malignancies, pre-conditioning with *in-vivo* lymphodepleting chemotherapy appears to be essential for significant expansion of infused cells probably related to the *in-vivo* induction of lymphocyte activating interleukins and depletion of regulatory T cells (37–39). Since in our study we scheduled to treat patients in remission in a prophylactic setting, no lymphodepleting conditioning could be applied. Secondly, we hypothesized that co-expression of the virus specific TCR and the HA-1H specific TCR-transduced T cells would lead to expansion when exposed to either viral antigens or the HA-1H antigen (34, 35). The *in-vivo* data suggest that in the absence of significant exposure to recipient-derived HA-1H antigen expressing hematopoietic cells, expansion of T cells co-expressing both TCRs did not or hardly occur. We hypothesize that by codon optimization and cysteine modification of the HA-1H TCR, and selection of virus specific T cells with a weak competitor phenotype we created an HA-1H TCR that successfully competed for membrane expression with the endogenous virus-specific TCR, resulting in lack of expansion in the presence of only viral antigens (34, 35, 40). This has likely resulted in the expansion of mostly the non-transduced virus-specific T cells from the infused product during viral reactivation after transplantation. As a consequence, in only 2 patients significant persistence and expansion of HA-1H TCR-transduced CMV or EBV-specific T cells could be illustrated, correlating with a viral reactivation. Only in the patient who showed smoldering relapse at the time of infusion, low frequencies of HA-1H TCR-expressing cells were present, but also under these circumstances, the malignant cells outgrew the HA-1H TCR-transduced CMV or EBV-specific T cells. There appeared to be no HA-1H antigen escape variant in this patient, since also the relapsed AML cells were shown to be recognized by HA-1H TCR-transduced CMV or EBV-specific T cells *ex-vivo*.

In one patient, lethal PTLD developed in the presence of EBV-specific T cells with specificities that were also present in the infused investigational product. Further analysis illustrated these EBV-positive clonal B cells of donor origin were transformed not to express the antigen specificities targeted by the circulating EBV-specific T cells. No significant numbers of B cells were present in the investigational product, and therefore we concluded that this complication was not due to the experimental treatment and that lack of control by the T cells present in the product was caused by an antigen negative variant which has been found more frequently in patients with monoclonal PTLD.

In conclusion, we have demonstrated that HA-1H TCR-transduced CMV or EBV-specific T-cell products can be reproducibly made if sufficient virus-specific T cells can be isolated from virus seropositive donors. Infusion of these products into patients with high-risk AML appears to be safe, but overall feasibility and efficacy of this approach appears to be too low to allow further development of this investigational product. A new strategy will be explored using products consisting of donor-originated CD8 T cells isolated from the patient after transplantation and transduced with the HA-1H TCR gene to be infused following immune conditioning in patients with persistent or relapsed hematological malignancies after HA-1H-mismatched transplantation. A new clinical trial has recently been approved (EudraCT 2019-002346-20) that implemented several improvement to the limitations of the strategy followed in this manuscript. The improvements aim to overcome the identified weaknesses of lack of lymphodepleting condition and lack of HA-1H antigen expression in the recipient. Transduction of not only virus-specific T cells but all donor derived CD8 T cells circulating in allogeneic transplanted patients and infusion of a targeted dose of HA-1H TCR transduced T cells will result in higher numbers of infused T cells. Again, HA-1H TCR transduction is preferred over using peptide stimulated HA-1H specific T cells since that method is much more time consuming and laborious without expected better efficacy. This will allow further evaluation of the potential efficacy of MiHA-TCR-transduced T-cell products in the treatment of hematological malignancies in the context of alloSCT.

## Data Availability Statement

The raw data supporting the conclusions of this article will be made available by the authors, without undue reservation, to any qualified researcher.

## Ethics Statement

The studies involving human participants were reviewed and approved by Centrale Commissie Mensgebonden Onderzoek (CCMO). The patients/participants provided their written informed consent to participate in this study. Written informed consent was obtained from the individual(s) for the publication of any potentially identifiable images or data included in this article.

## Author Contributions

PB, IJ, ML, HV, JF, and MH designed the research, analyzed results, and wrote the paper. ML, RB, HE, RH, CH, SV, LH, PL, J-JZ, PM, and IJ performed experiments and were responsible for manufacturing the T-cell products. All authors contributed to the article and approved the submitted version.

## Conflict of Interest

The authors declare that the research was conducted in the absence of any commercial or financial relationships that could be construed as a potential conflict of interest.
