# Supplementary material for: HA-1H T-Cell Receptor Gene Transfer to Redirect Virus-Specific T Cells for Treatment of Hematological Malignancies After Allogeneic Stem Cell Transplantation: A Phase 1 Clinical Study
Source: Front Immunol. 2020 Aug 20;11:1804. doi: 10.3389/fimmu.2020.01804 (PMC7468382; doi:10.3389/fimmu.2020.01804)
Supplement: Supplementary file 1 [file Data_Sheet_1.PDF]

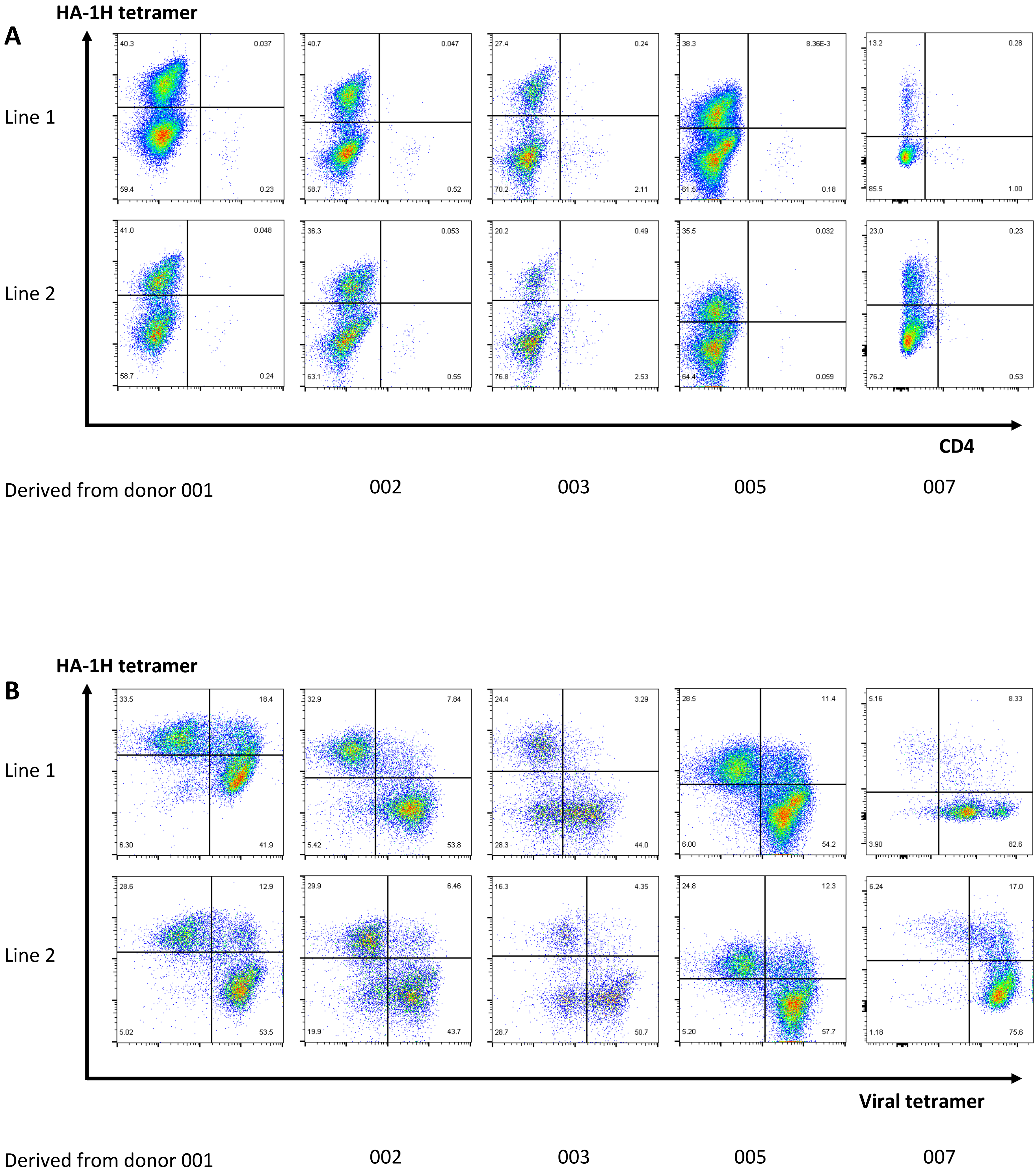

Supplemental Figure 1. Composition of the infused cell products. **A.** Flowcytometric analysis of first and second cell line. Percentage of HA-1H tetramer positive cells within the CD4 negative fraction is shown in the left upper quandrant. Table 1 showed that all products contained >96% T cells. **B.** HA-1H tetramer stained against the combination of used viral tetramers, as listed in Table 1.
